# Vortex lattices of layered HTSCs at different vortex–vortex interaction potentials

**DOI:** 10.3762/bjnano.16.27

**Published:** 2025-03-13

**Authors:** Valerii P Lenkov, Anastasia N Maksimova, Anna N Moroz, Vladimir A Kashurnikov

**Affiliations:** 1 National Research Nuclear University MEPhI, Moscow, 115409 Russia

**Keywords:** high-temperature superconductor, HTSC, intertype superconductors, Monte Carlo method, vortex lattice, vortex–vortex interaction potential

## Abstract

Magnetization reversal processes in a vortex system with different potentials of vortex–vortex interaction were studied using the Monte Carlo method within the framework of a two-dimensional model of a layered high-temperature superconductor. Interaction potentials close to the potential applicable in superconductors with the Ginzburg–Landau parameter κ = 1/2 (intertype superconductors) and in ferromagnetic superconductors have been analyzed. Clustering of the vortex system is demonstrated. The melting of a vortex lattice with increasing temperature has been studied.

## Introduction

Type-II superconductors, as shown by numerous studies, have a complex phase diagram in a magnetic field. In fields greater than the first critical field *H*_c1_ and less than the second critical field *H*_c2_, at temperatures below the critical temperature the superconductor is in a mixed state, in which the magnetic field penetrates the superconductor in the form of Abrikosov vortices [1]. In high-temperature superconductors (HTSCs), such as Y- and Bi-based cuprates, the vortex lattice is further complicated since these compounds have a layered structure [2–6]. The vortex filament in these superconductors can be represented as a stack of pancakes, that is, flat vortices located in the CuO planes and connected by Josephson interaction through interplanar gaps. The vortex structure in layered HTSCs is still a subject of research. In [7], it was shown that Pb doping increases the two-dimensional ordering of the pancakes, which also enhances the pinning of vortices on defects. In [8], the vortex system in a HgBa_2_CuO_4+δ_ monocrystal was studied. The measurements were performed in a wide range of temperatures and magnetic fields, and the phase diagram of the vortex system was obtained as a result of the measurements. The vortex system was studied by measuring the magnetization of the sample. The relaxation rate of the magnetization caused by the process of thermally activated creep of the magnetic flux was also measured. In this case, the phenomenon of collective creep of vortex bundles was discovered. Indications were also obtained that the second peak on the magnetization curve (second magnetization peak) at low temperature (less than 0.55*T*_c_) coincides with the transition between the regimes of the flow of the vortex lattice. The measurements were also performed in a magnetic field inclined with respect to the superconducting planes.

The phase diagram of the vortex system becomes even more complex in an inclined magnetic field. As studies [9–11] have shown, the structure of the vortex lattice depends on the anisotropy parameter of the superconductor in the form of the Josephson length λ_J_ = γs, where *s* is the distance between the superconducting planes, γ is the anisotropy parameter, and λ is the London penetration depth of the magnetic field. When the ratio λ/λ_J_ *<* 0.46, crossed lattices of Abrikosov and Josephson vortices (vortex chains) are observed; at λ/λ_J_ *>* 0.46, the Josephson vortices disappear, and a regular lattice of inclined Abrikosov vortices remains. In the first case, Josephson vortices can impart a zigzag shape to Abrikosov vortices; this shape, as shown in [9–11], leads to effective attraction of vortex filaments. At close distances (less than λ), short-range repulsion is still preserved, and the repulsion is also preserved at large distances (5–10λ) between vortex centers. As a result, the potential takes a shape characterized by one minimum and one fairly flat maximum [12–14]. The vortex lattice in an inclined magnetic field has also been studied in [15–17].

The vortex–vortex interaction potential, different from the classical one typical in type-II superconductors *K*_0_(*r*/λ) (*r* is the distance between the vortex centers) [18], is also observed in superconductors characterized by the Ginzburg–Landau parameter 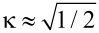. A vortex structure is also observed in such a superconductor; however, the interaction of the vortices is characterized by short-range attraction and long-range repulsion [19–21]. This form of the potential is characterized by the formation of clusters containing several vortices up to several dozen vortices. In [19], a molecular dynamics simulation of a vortex system in a superconductor with 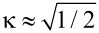 was performed, and a phase *B*–*T* diagram was obtained (*B* is the magnetic field and *T* is the temperature of the vortex system), which contains regions of a hexagonal vortex lattice, a striped structure, and a lattice of vortex clusters.

A potential different from the classical one is observed for intervortex interaction and in the so-called ferromagnetic superconductors [22]. The polarization of the magnetic moments of the crystal lattice induced by vortices leads to an interaction potential that has a minimum and a maximum (at distances greater than 10λ, repulsion is observed). In this work, the formation of vortex clusters and their motion under the action of the Lorentz force were investigated. Since their interaction potential is similar to intertype, our results are applicable to them. A description of the mechanism of the emergence of ferromagnetic and superconducting subsystems, as well as experimental confirmation of clustering can be found in [23]

Vortices in type-II superconductors can form clusters under certain conditions, which affects the phase diagram of the superconductor and, consequently, its magnetic and transport properties. Therefore, it is of interest to study the magnetization reversal processes in a sample under conditions that allow for vortex clustering. Additionally, the process becomes more complicated in the presence of pinning centers. An effective method for research is the Monte Carlo method for a vortex system. The aim of this work is to study the magnetization and vortex configurations in a vortex system with different intervortex interaction potentials. Magnetization curves have been calculated, vortex configurations in a sample have been obtained and analyzed, the intervortex interaction potential of which is identical in shape to the interaction potential in ferromagnetic superconductors. The modeling results can also be useful for analyzing vortex configurations in a layered anisotropic HTSC in an inclined magnetic field.

## Methods

Calculations were performed using the Monte Carlo method within the framework of a two-dimensional model of a layered HTSC [24–26]. Most of the HTSCs applicable in practice are strongly anisotropic substances characterized by the anisotropy parameter γ *>* 10. Examples of such HTSCs are YBa_2_Cu_3_O_7−δ_ (γ ≈ 10) and Bi_2_Sr_2_CaCu_2_O_8−δ_ (γ ≈ 200) [1]. Such superconductors have a layered structure and can be modeled as a stack of superconducting planes separated by an insulating gap. The Abrikosov vortex in such a structure can be represented as a stack of flat layered vortices, the so-called pancakes, connected by an interplanar bond. In samples that do not have artificial pinning centers, or in samples with columnar defects perpendicular to the superconducting planes, the average deviation of pancakes from the axis of the vortex filament due to thermal motion is much less than λ, that is, the London penetration depth of the magnetic field into the superconductor [27]. Therefore, vortex filaments are approximately straight, and only one HTSC layer can be considered for modeling. A vortex system at a temperature not too close to the critical temperature can be modeled as an ensemble of particles interacting with a long-range potential. Then the energy of the vortex system can be represented as follows:


[1] $$G=\sum_{i}\left(\frac{1}{2}\sum_{j\neq i}U_{ij}+\varepsilon +U_{\text{h}}\right).$$

The first term corresponds to the pairwise interaction of pancake vortices belonging to one HTSC layer. For Abrikosov vortices in a superconductor with κ ≫ 1, the interaction potential between vortices has the form




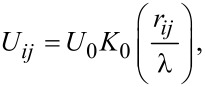




where




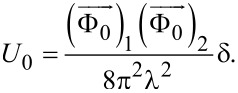




Here, δ is the thickness of the superconducting layer, *K*_0_ is the zeroth-order Macdonald function, 
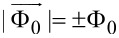
, where the sign is chosen depending on the sign of the field that generated the vortex, and Φ_0_ is the magnetic flux quantum. The second term corresponds to the total self-energy of the vortices in the HTSC layer. The indices *i* and *j* number the pancakes in the layer under consideration,




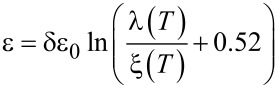




is the vortex self-energy per superconducting layer with




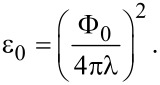




The simulation is performed for a vortex lattice in a sample whose size in the plane of the superconducting layer is 5 × 5 μm. To eliminate the influence of the boundary, the simulation region has periodic boundary conditions along both coordinate axes. Under the conditions of this geometry, the third term in Equation 1, corresponding to the additional vortex energy associated with the external magnetic field, has the form




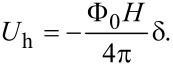




The calculation also takes into account the temperature dependence of the London magnetic field penetration depth and the coherence length [28]. In this paper, the following forms of this dependence are used:




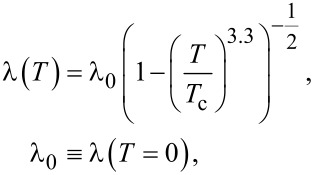




and




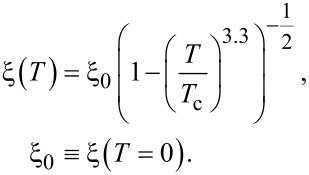




These relations fairly accurately reflect the temperature dependence of the characteristic lengths in bismuth HTSC [29].

In this work, in addition to the classical potential that describes the interaction of Abrikosov vortices, two more types of potential were analyzed. The first model interaction potential corresponds to superconductors with the Ginzburg–Landau parameter 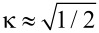 and can be written as [14]


[2] $$U\left(r\right)=\left(-q\right)\left(\ln\frac{r}{r+\lambda }+k\exp\left(-\frac{r}{\lambda }\right)\right).$$

**Figure 1:**
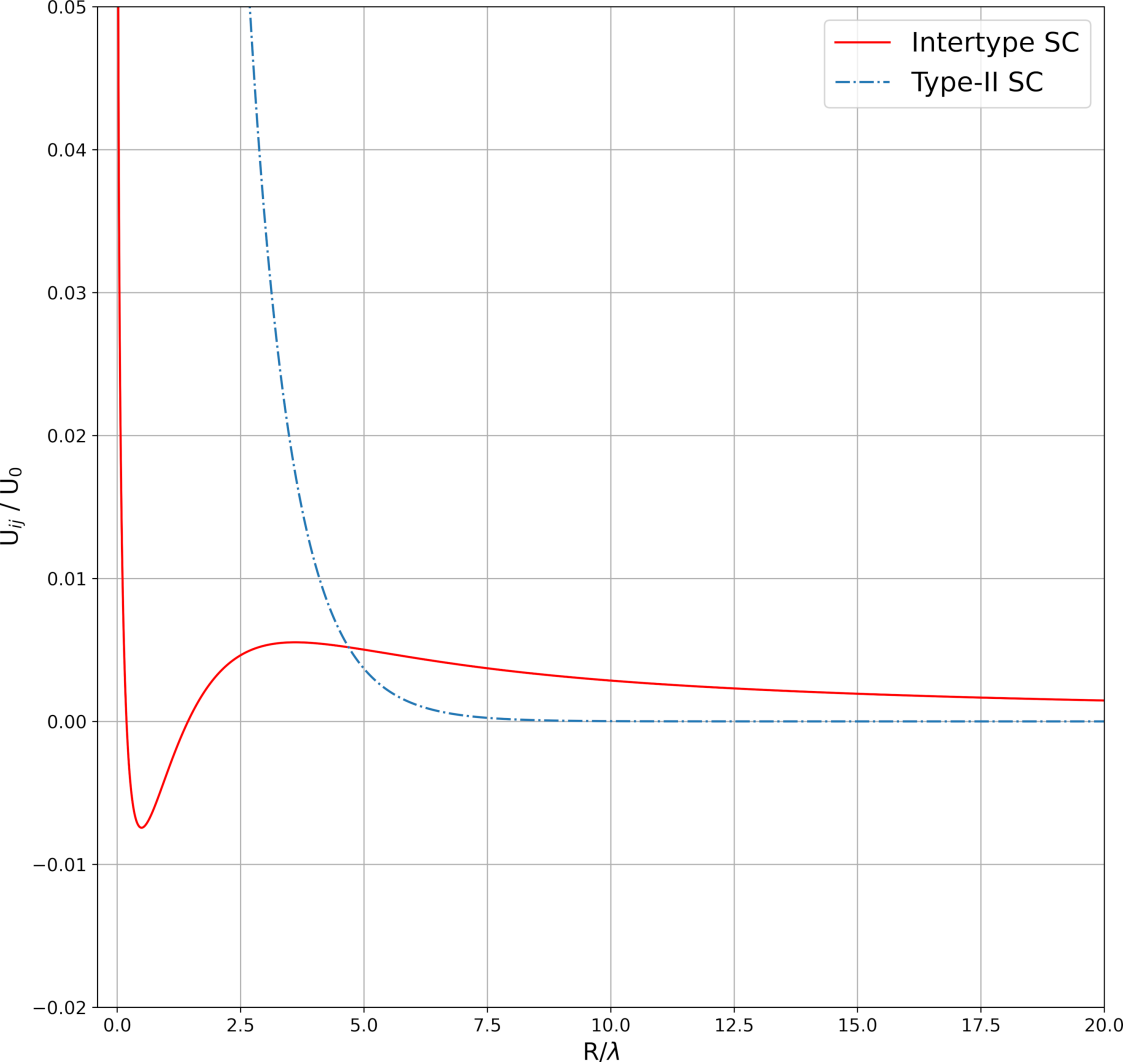
Model potential from Equation 2.

A typical form of this potential is shown in Figure 1.

**Figure 2:**
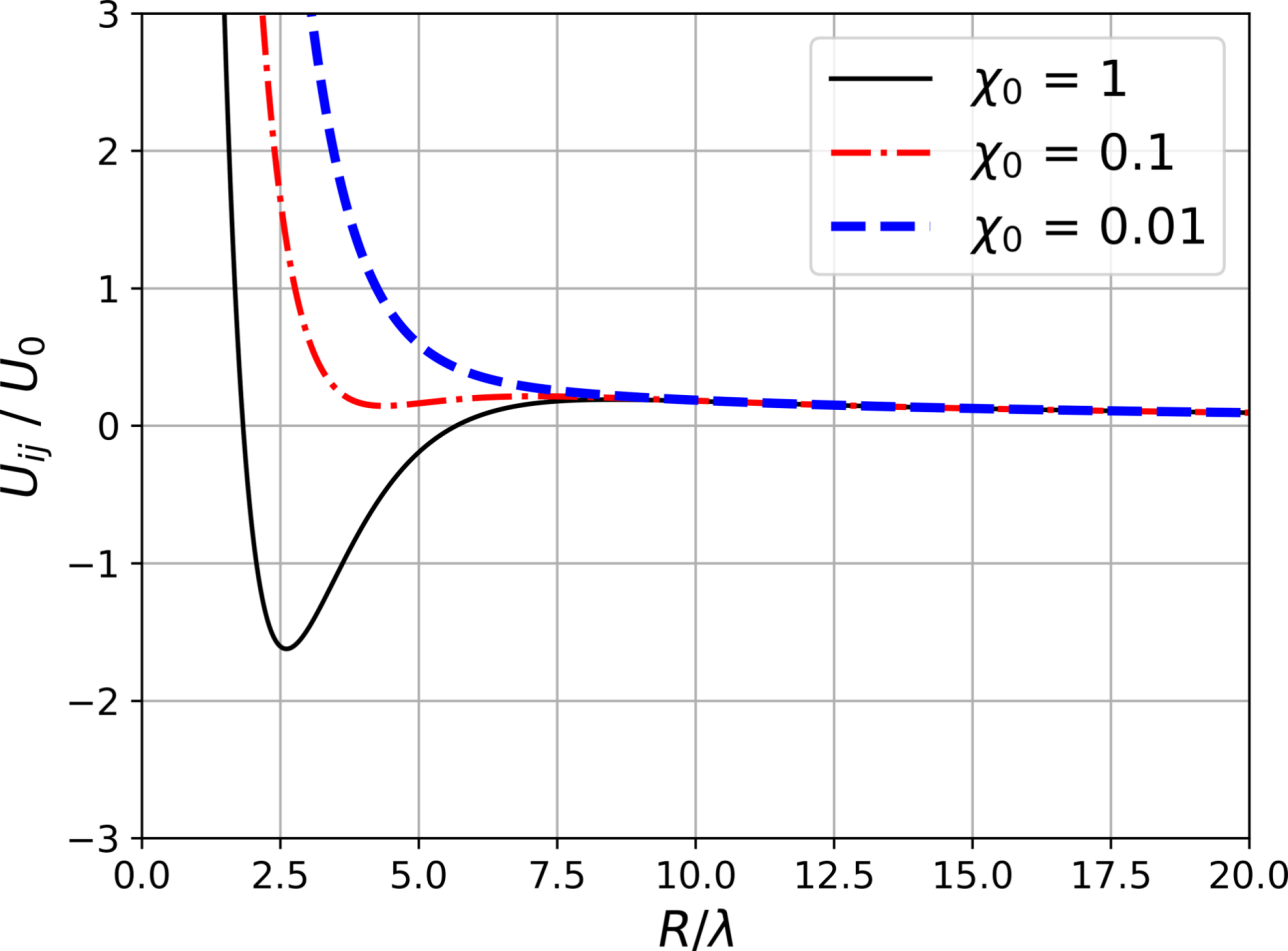
Potential from Equation 3 at different values of magnetic susceptibility.

Another potential of vortex–vortex interaction investigated in the work corresponds to the interaction of vortices in a ferromagnetic superconductor [22]. This potential can be written as a linear combination of the potentials *U*_r_(*r*) and *U*_a_(*r*), responsible for long-range repulsion and short-range attraction, respectively:


[3] $$\begin{array}{c} U_{\text{r}}(r)=\frac{\Phi_{0}^{2}\delta }{8\pi^{2}\lambda_{\text{e}}^{2}}K_{0}\left(\frac{r}{\lambda_{\text{e}}}\right)+\frac{\Phi_{0}^{2}}{8\pi \Lambda }\left[H_{0}\left(\frac{r}{\Lambda }\right)-Y_{0}\left(\frac{r}{\Lambda }\right)\right],\\ U_{\text{a}}(r)=-\frac{\delta \Phi_{0}^{2}\chi_{0}r}{4\pi \left(1+4\pi \chi_{0}\right)\lambda_{\text{e}}^{3}}K_{1}\left(\frac{r}{\lambda_{\text{e}}}\right), \end{array}$$

where Λ = 2λ_e_coth(δ/λ_e_) is the modified Pearl length,




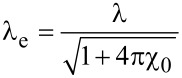




is the magnetic field penetration depth after renormalization, and χ_0_ is the magnetic susceptibility of the material. The higher the magnetic susceptibility, the greater the difference between the model potential and the potential from Equation 1 and the more pronounced is the tendency of vortices to form clusters (Figure 2).

Both considered model potentials have one distinctive minimum and a weakly defined maximum. The energy minimum for the potential from Equation 2 corresponds to a distance of less than λ between the vortex centers. As further calculations show, this leads to the formation of clusters, the number of vortices in which can reach 100. The shape of the potential from Equation 3 depends significantly on the value of the magnetic susceptibility of the superconductor, χ_0_. With an increase in χ_0_, the depth of the minimum increases; starting from a certain value, the minimum disappears. For such a potential, the number of vortices in a cluster turns out to be up to several dozens. At large distances, both potentials monotonically tend to 0. The numerical coefficients in the potentials from Equation 2 and Equation 3 are selected in such a way that the numerical values of the potential at large distances approximately correspond to the potential from Equation 1.

In the calculations it was assumed that λ(0) = 180 nm and ξ(0) = 2 nm. The critical temperature was taken to be 84 K. The first critical field with this choice of parameters is *H*_c1_ = 290 G. These values correspond to the experimental values for yttrium- and bismuth-based cuprates [1]. With these parameters and in magnetic fields not exceeding 1000 G, no more than 1000 vortices are generated in the sample. For the specified values of the superconductor parameters, a model problem was solved: The vortex lattice structure was calculated under the conditions of the intervortex interaction potential being different from Equation 1. When changing the values of ξ, λ, and *T*_c_, the calculation results will change quantitatively, but the observed effects are qualitatively preserved. The obtained results can be useful for designing superconducting devices of micrometer and submicrometer size. For the potential from Equation 2, vortex lattice melting with increasing temperature was studied. For the potential from Equation 3, the formation of a vortex lattice was investigated for different values of magnetic susceptibility χ_0_.

In Monte Carlo(MC) simulations, the thermalization of the system occurs quite quickly, with the energy plateauing after about 10,000 MC steps. However, subsequent calculations typically used 200,000 steps of the algorithm; statistics were collected based on the last 100,000 steps. Vortex configurations were calculated after the energy graph reached a plateau.

## Results and Discussion

**Figure 3:**
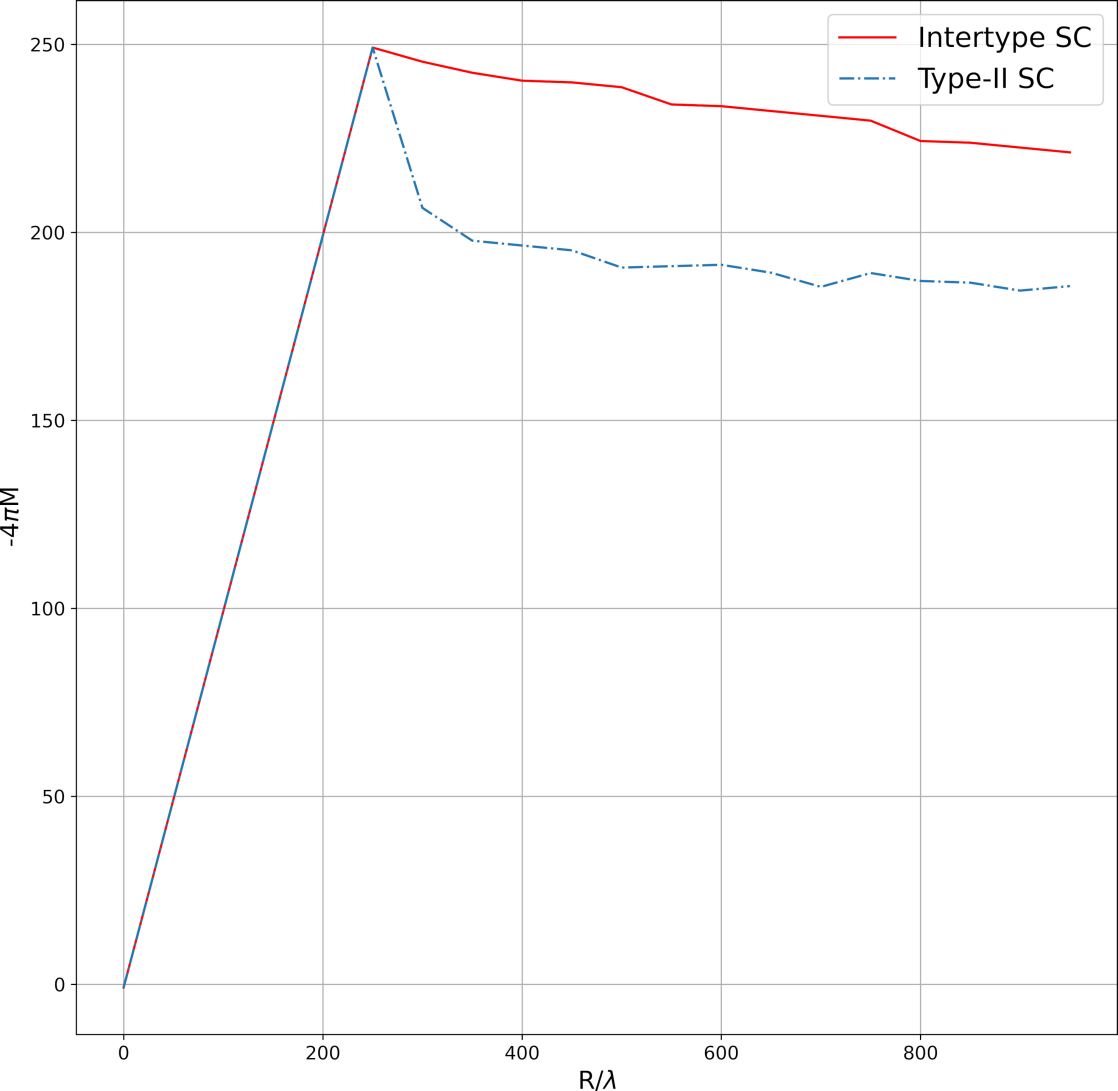
Magnetization curves at the interaction potentials from Equation 1 and Equation 2.

In most known type-II superconductors (e.g., in cuprates), in magnetic fields much lower than *H*_c2_, the interaction between vortices is described by the expression in Equation 1. The vortex structure under these conditions is a hexagonal lattice. Figure 3 shows the magnetization curves of a superconducting sample calculated under the assumption that the vortices interact via the potential from Equation 1 (dashed curve) and via the potential from Equation 2 (solid curve). The magnetic field increased from 0 to 1000 G. In both cases, the magnitude of the first critical field *H*_c1_ is the same. After the first critical field, the magnetization curve for the potential from Equation 2 is higher than for the potential from Equation 1. This effect can be easily explained using Figure 1. The interaction energy corresponding to the model potential at distances between vortices greater than 5λ exceeds the interaction energy corresponding to the classical potential from Equation 1, which leads, accordingly, to a stronger repulsion of the vortices.

**Figure 4:**
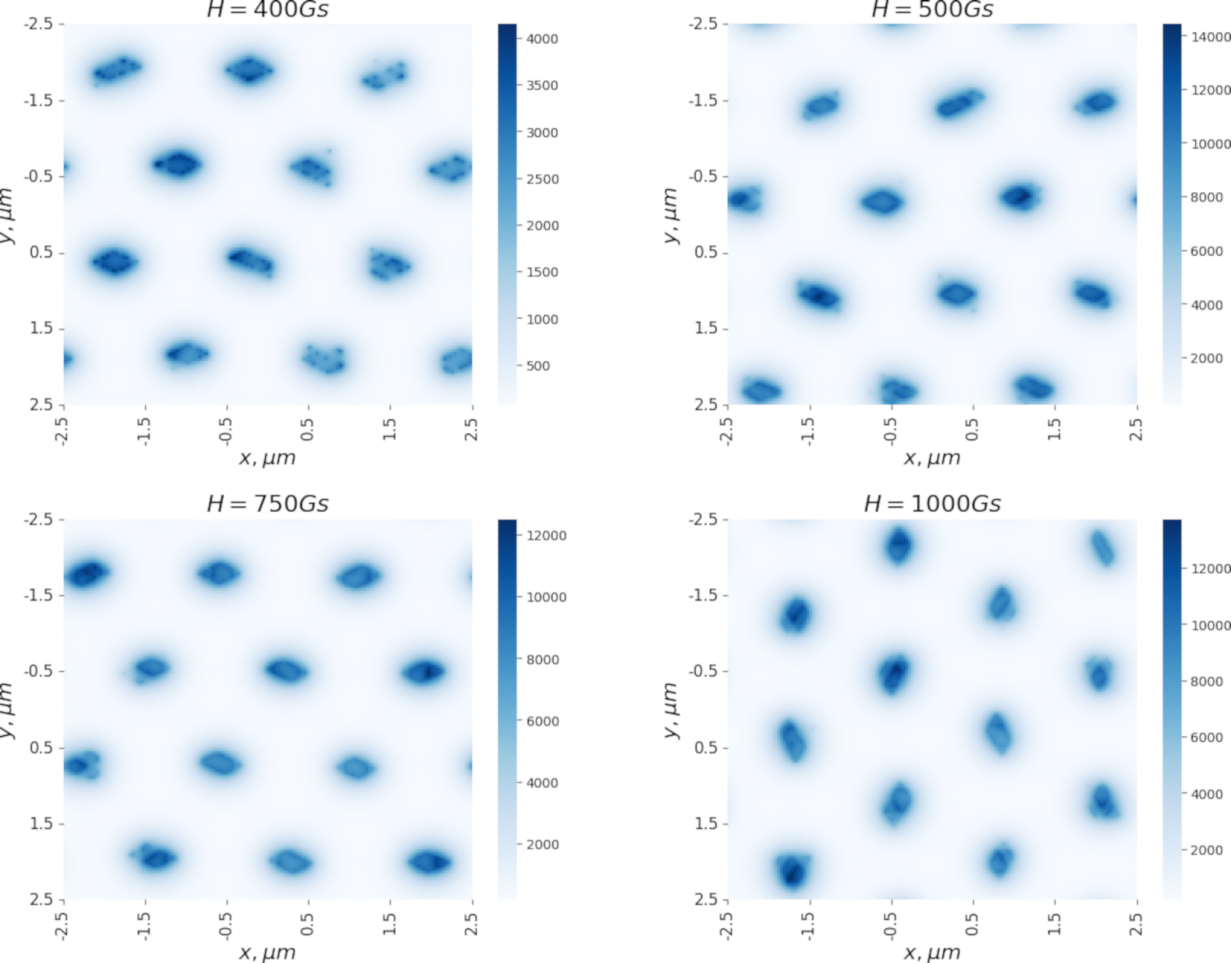
Magnetic field distribution in the sample at *T* = 1 K. Vortex configurations arising along the magnetization curve are shown.

It is of interest to analyze the vortex configurations arising during magnetization in a sample, the interaction between vortices in which is described by the model potentials from Equation 1 and Equation 2. Figure 4 shows the vortex configurations (magnetic field distribution in the sample) arising along the magnetization curve (Figure 3). Since the potential from Equation 2 has an energy minimum at a distance of approximately λ/4 between the vortex centers, the formation of vortex clusters should be expected. Indeed, Figure 4shows regularly shaped clusters consisting of several dozen vortices. Because of long-range repulsion, individual clusters form a hexagonal lattice. At a temperature of 1 K, the clusters have the shape of rhombuses. The peculiarity of the calculated vortex configurations is easily seen from Figure 4. As the magnetic field increases, the number of clusters in the sample remains unchanged, and new vortices generated in the sample join the existing clusters. The number of vortices in a cluster changes from 17–20 at *H* = 400 to 80 at *H* = 1000 G.

**Figure 5:**
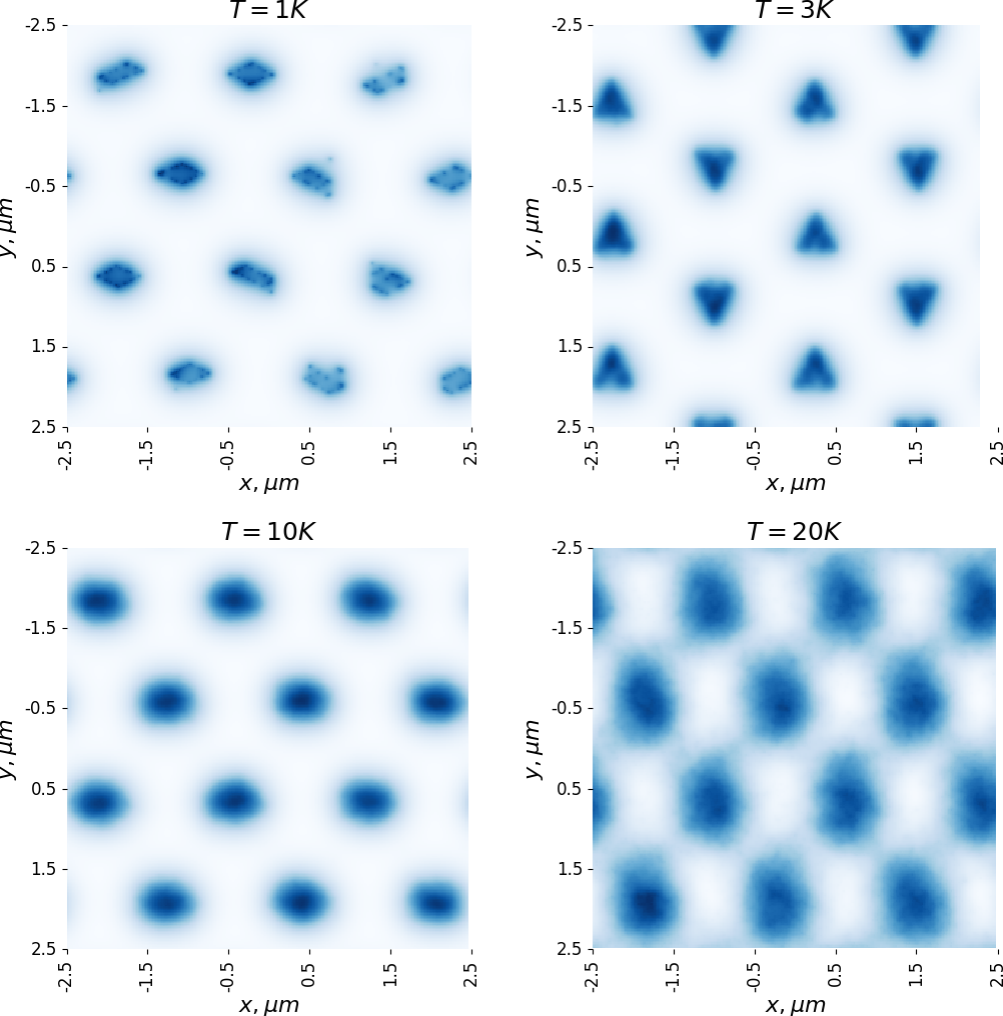
Magnetic field distributions in a sample at various values temperature; magnetic field *H* = 400 G.

**Figure 6:**
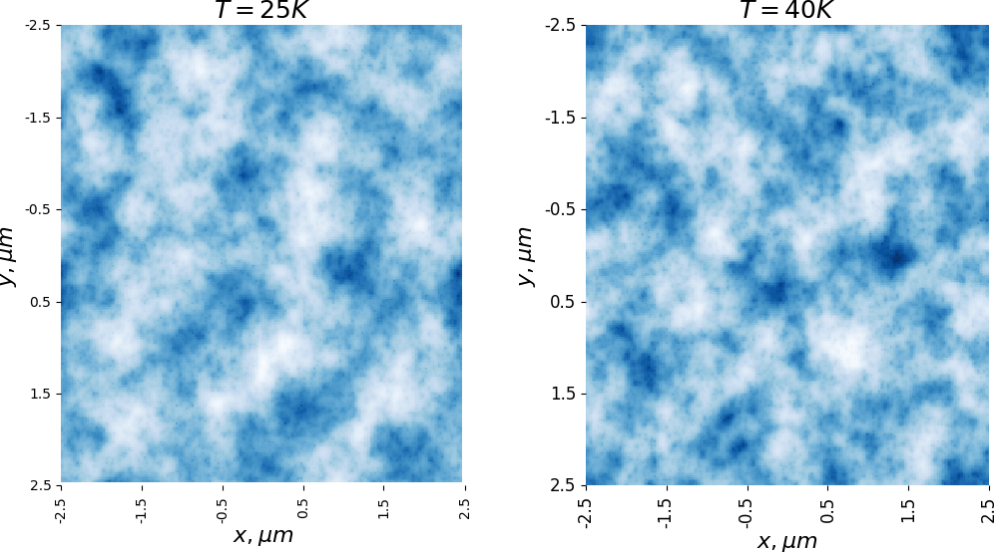
Melting of a vortex lattice with increasing temperature; magnetic field *H* = 400 G.

It is also of interest to study the behavior of the vortex lattice with changing temperature. The magnetization curves (Figure 3) were calculated at *T* = 1 K. For further analysis, a vortex configuration in a magnetic field of *H* = 400 G was chosen. Figure 5 and Figure 6 show the distributions of the magnetic field in the sample with increasing temperature. It is evident that even an increase in temperature to 3 K leads to noticeable thermal motion of the vortices, blurring the boundaries of the clusters (in all figures, the distribution of the magnetic field is calculated based on the averaged configuration of the vortices). It should be noted that the intensity of the thermal motion of the vortices increases only inside the clusters. Thermal motion of the clusters themselves does not occur in the temperature range from 1 to 20 K. However, a further increase in temperature leads to the destruction of the ordered lattice of clusters (Figure 6, which shows the magnetic field distributions at 25 and 40 K). We also note the change in the shape of the clusters when moving from 1 to 3 K (Figure 5).

**Figure 7:**
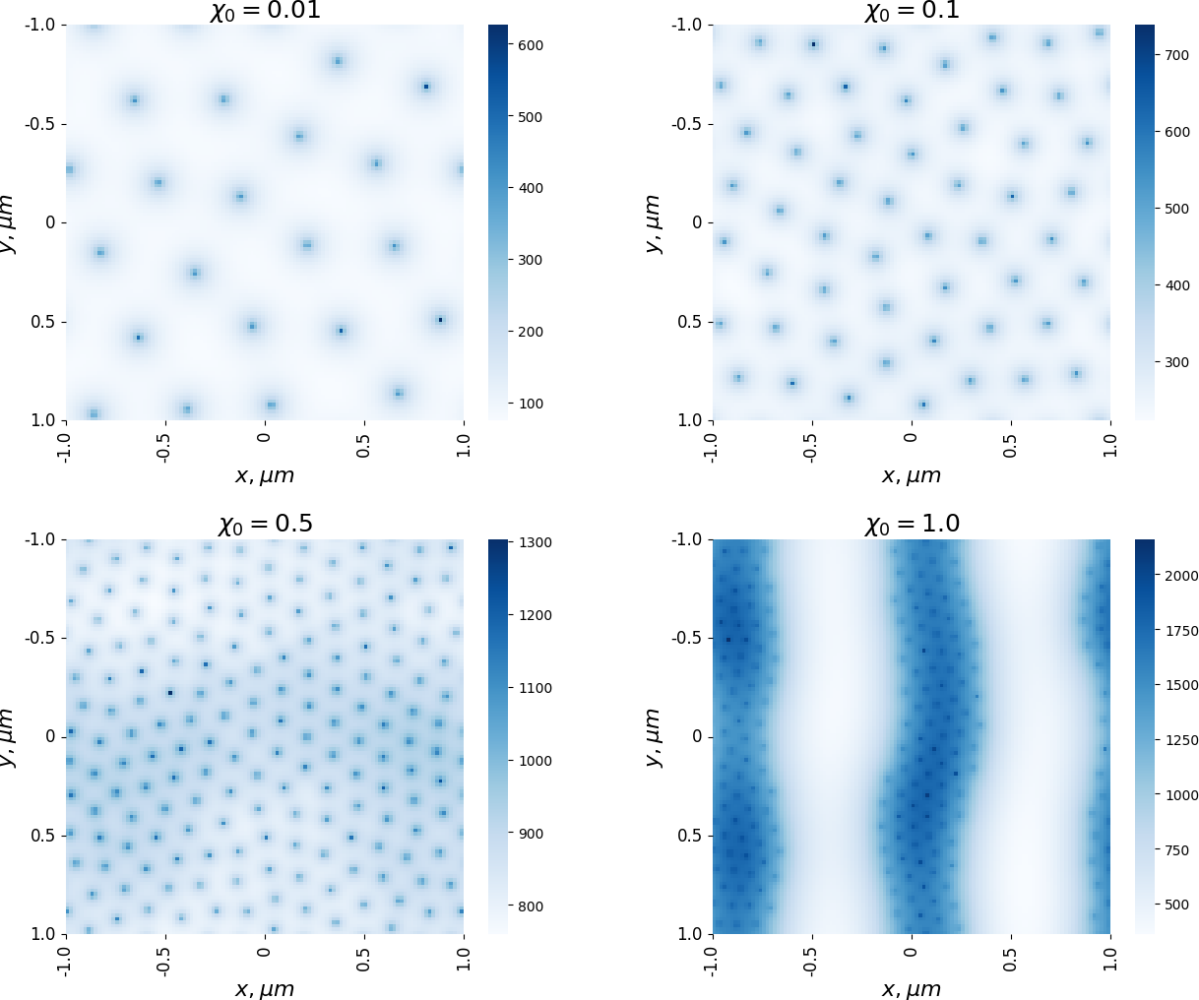
Magnetic field distribution for a sample with the vortex–vortex interaction potential from Equation 3 at different values of magnetic susceptibility χ_0_.

**Figure 8:**
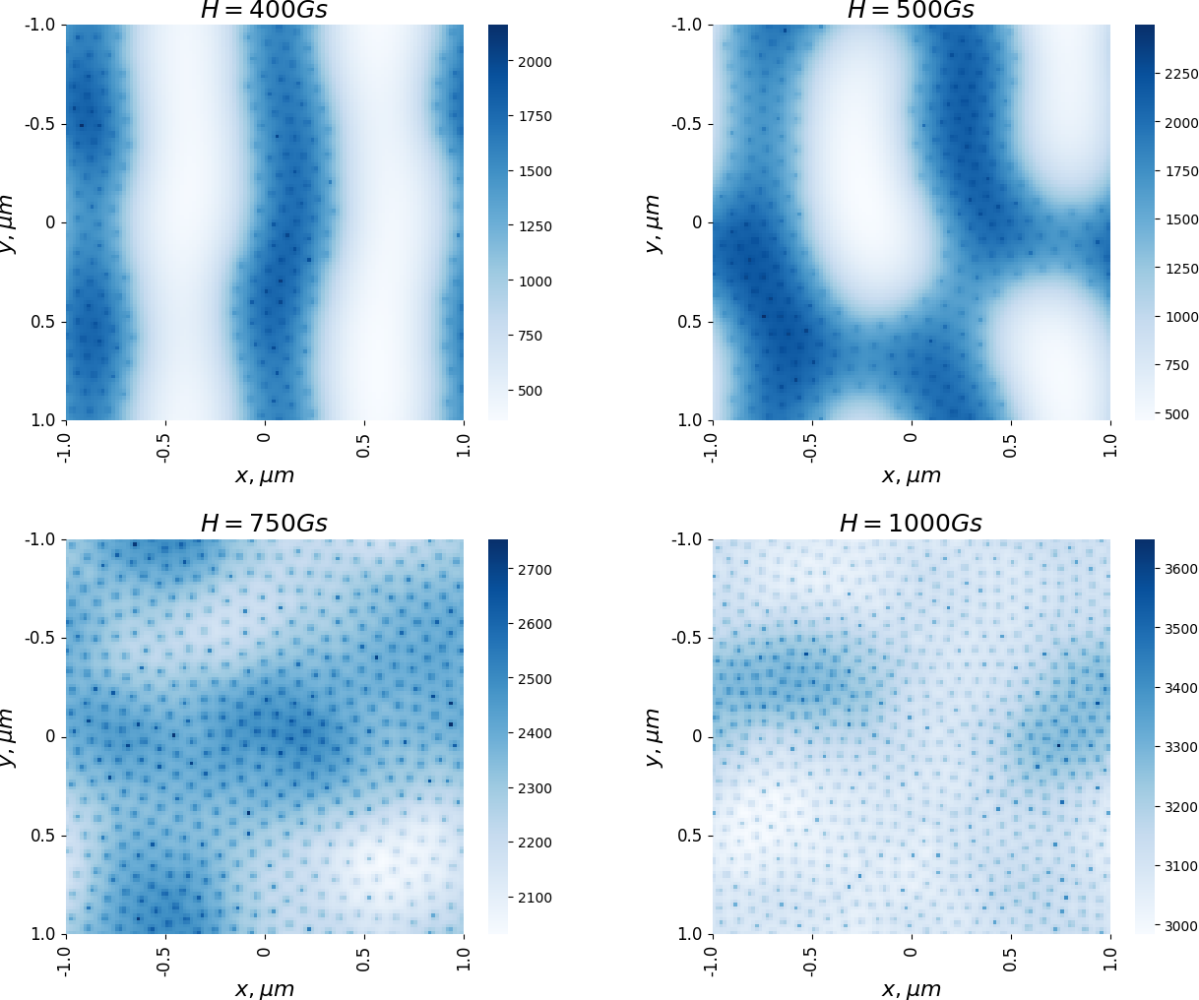
Magnetic field distribution in the potential from Equation 3 at different field values; χ_0_ = 1.0.

The vortex configurations for the model potential from Equation 3 were simulated in a similar manner. The vortex configurations calculated for different values of the magnetic susceptibility are shown in Figure 7. At χ_0_ = 0.01, the ferromagnetic part of the potential is small compared to the terms responsible for the intervortex repulsion, and clusters are not formed. At low values of the magnetic susceptibility, the vortices form a hexagonal lattice. As χ_0_ increases to 0.5, the process of vortex clustering begins, which can be seen as a darker region in the middle of the sample in Figure 7. At χ_0_ = 1, strip-shaped clusters are formed. The vortices inside the clusters still form a hexagonal lattice. In Figure 8, vortex configurations arising in the sample with increasing magnetic field are presented. Increasing the magnetic field leads to the birth of new vortices, which, unlike the situation with the potential from Equation 2, are not added to the existing clusters, but a noticeable restructuring of the vortex lattice occurs. If at *H* = 400 G individual clusters in the form of stripes are visible, then already at *H* = 750 G the vortices occupy the entire sample, and the clusters are replaced by regions with a higher average vortex distribution density. It should be noted that clusters in the form of stripes were observed in the numerical simulation in [19].

## Conclusion

Within the framework of a two-dimensional model of a layered HTSC, the configurations of the vortex lattice were simulated under conditions where the intervortex interaction potential differs from the usual one observed in type-II superconductors. The shape of the potential corresponds to the potential observed in superconductors with the Ginzburg–Landau parameter 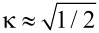 and the interaction potential observed in ferromagnetic superconductors. Clustering of vortices was observed in magnetic fields from 400 to 1000 G. For a vortex system interacting with a potential characteristic of intertype superconductors, melting inside vortex clusters was observed with increasing temperature. At temperatures of 1–10 K, a hexagonal lattice of clusters is observed; upon reaching 20–25 K, the cluster structure is no longer observed. For the interaction potential characteristic of ferromagnetic superconductors, pronounced clustering is shown at fields near *H*_c1_. The difference between the results obtained and those given in the literature is in the consideration of the magnetization reversal process and the study of the temperature effect. The calculation results can be useful in designing various superconducting devices.

## Data Availability

Data generated and analyzed during this study is available from the corresponding author upon reasonable request.
